# Agonist triggering in oocyte donation programs—Mini review

**DOI:** 10.3389/fendo.2022.838236

**Published:** 2022-08-26

**Authors:** Robert Najdecki, Georgios Michos, Nikos Peitsidis, Evangelia Timotheou, Tatiana Chartomatsidou, Stelios Kakanis, Foteini Chouliara, Apostolos Mamopoulos, Evangelos Papanikolaou

**Affiliations:** ^1^ Assisting Nature, Centre of Assisted Reproduction and Genetics, Thessaloniki, Greece; ^2^ 3rd Department of Obstetrics and Gynecology, Aristotle University of Thessaloniki, Thessaloniki, Greece

**Keywords:** agonist triggering, oocyte donation programs, OHSS-free programs, optimal dose for agonist triggering, impact on acceptor’s pregnancy outcome

## Abstract

Oocyte donation programs involve young and healthy women undergoing heavy ovarian stimulation protocols in order to yield good-quality oocytes for their respective recipient couples. These stimulation cycles were for many years beset by a serious and potentially lethal complication known as ovarian hyperstimulation syndrome (OHSS). The use of the short antagonist protocol not only is patient-friendly but also has halved the need for hospitalization due to OHSS sequelae. Moreover, the replacement of beta-human chorionic gonadotropin (b-hCG) with gonadotropin-releasing hormone agonist (GnRH-a) triggering has reduced OHSS occurrence significantly, almost eliminating its moderate to severe presentations. Despite differences in the dosage and type of GnRH-a used across different studies, a comparable number of mature oocytes retrieved, fertilization, blastulation, and pregnancy rates in egg recipients are seen when compared to hCG-triggered cycles. Nowadays, GnRH-a tend to be the triggering agents of choice in oocyte donation cycles, as they are effective and safe and reduce OHSS incidence. However, as GnRH-a triggering does not eliminate OHSS altogether, caution should be practiced in order to avoid unnecessary lengthy and heavy ovarian stimulation that could potentially compromise both the donor’s wellbeing and the treatment’s efficacy.

## Introduction

Oocyte donation is a fundamental procedure for many women and *in vitro* fertilization (IVF) laboratories as the number of couples seeking such treatment is steadily increasing. Based on the latest European IVF-monitoring Consortium research, egg donation treatments represent about 32.4% of all assisted reproductive technology (ART) cycles in European countries (1). These highly complicated programs, in which very young women undergo heavy ovarian stimulation protocols, were for many years beset by a serious and potentially lethal complication known as ovarian hyperstimulation syndrome (OHSS). OHSS is characterized by extravasation of fluids into the third space due to increased vascular permeability mediated by vasoactive factors released by stimulated ovaries when exposed to beta-human chorionic gonadotropin (b-hCG) leading to effusions and vascular dehydration. Clinically, symptoms can be classified from mild to severe, ranging from mild abdominal discomfort and nausea to breathlessness, affected renal/liver function, and thromboembolic events.

In recent years, the innovative gonadotropin-releasing hormone (GnRH) antagonist has made it possible for us to apply new protocols, patient-friendly and safer, in the field of assisted reproduction. Implementing the antagonist protocol alone has significantly decreased the incidence of severe OHSS and the need for hospitalization by 50% (2, 3).

Nevertheless, OHSS was still present in young women with good ovarian response (such as donors). Therefore, the need for an even safer therapeutic approach led the scientific community to opt for oocyte maturation triggering with GnRH agonists (GnRH-a) rather than the use of classical human chorionic gonadotropin (hCG). The use of GnRH-a triggering in these cases has almost eliminated any hyperstimulation complication, changing risky, old procedures of long agonist stimulation protocols into modern, friendly, and safe short antagonist treatments with GnRH-a triggering (4). This modification has really brought about significant changes in IVF cycles and oocyte donation programs in particular (4). The donation programs are an excellent field for utilizing GnRH-a-induced maturation, as its luteolytic effect is not detrimental, since the donor herself will not undergo fresh embryo transfer.

The current mini review aims to review all available literature related to GnRH-a ovulation triggering in oocyte donation cycles in terms of dose administrated, OHSS occurrence, oocyte number, and embryo quality additionally to recipient pregnancy outcomes.

## Materials and methods

The present mini review, however, followed the reporting recommendations of the Preferred Reporting Items for Systematic Review and Meta Analyses (PRISMA) statement. Two authors (TC and ET) searched the electronic databases PubMed (MEDLINE) and CENTRAL covering the period from 1990 until December 2021. The reference list of all studies was scanned. Studies had to fulfill the following eligibility criteria: oocyte donation stimulated cycles, GnRH-antagonist protocol, OHSS incidence, type and dose of GnRH-a triggering, oocyte number, and embryo quality. Three authors (RN, GM, and EP) independently screened each of the identified studies based on the predefined eligibility criteria. Any disagreements were resolved by discussion.

The literature search yielded 110 studies. The titles and abstracts were screened, and the full text of 22 studies was evaluated. Seven studies fulfilled the inclusion criteria for the current review (**Table 1**).

**Table 1:** Special characteristics of the included studies.

Study	Study design	Patients type	OHSSrisk	Agonist used	Type of protocol/gonadotropins
**Acevedo, 2006** (5)	RCT	Donors	High	Triptorelin 0.2 mg	Antagonist fixed/recFSH/HMG
**Galindo, 2009** (6)	RCT	Donors	High	Triptorelin 0.2 mg	Antagonist fixed/recFSH
**Melo, 2009** (7)	RCT	Donors	High	Triptorelin 0.2 mg	Pill/antagonist fixed/recFSH
**Sismanoglu, 2009** (8)	RCTcrossover	Donors	High	Leuprolide 0.15 mg	Antagonist flexible/recFSH
**Bodri, 2009** (9)	Retrospective	Donor	High	Triptorelin 0.2 mg	Antagonist flexible/recFSH
**Streda, 2011** (10)	Prospective crossover	Donors	High	Triptorelin 0.2 mg	Antagonist/recFSH/HMG
**Tsakiridis, 2020** (11)	Prospective	Donors	High	Triptorelin 0.3 mg	Antagonist fixed D6/corifollitropin-alpha

### Pathophysiology of ovarian hyperstimulation syndrome in stimulated cycles and rationale for using agonist triggering instead of human chorionic gonadotropin triggering

GnRH was isolated in the early 1970s by the Nobel laureates Roger Guillemin and the group of Andrew V. Schally. It was one of the earliest well-characterized releasing hormones of the hypothalamic axis. GnRH consists of 10 amino acids. It is produced in the hypothalamus during the physiological menstrual cycle and periodically released to specific gonadotropin receptors located in the anterior pituitary (12).

In humans, in natural cycles, rapidly rising estradiol levels, through a positive feedback, increase GnRH levels by permissive mechanism and therefore gonadotropin secretion [follicle-stimulating hormone (FSH) and luteinizing hormone (LH)] inducing ovulation in women.

ιn IVF-stimulated cycles, supraphysiological estradiol levels cause untimely LH surges necessitating LH suppression in order to avoid premature ovulation. For many years, the long agonist protocol was the gold standard. The GnRH-a was used for LH downregulation, thus, agonist triggering was impossible to be implemented, as the receptors are already desensitized. Therefore, hCG was opted as a well-known substitute for the pre-ovulatory LH surge, presenting a very similar biochemical behavior as the urinary purified or recombinant form (13, 14).

However, OHSS remained the most serious complication of ovarian stimulation in ART (15). The presence of hCG is the basic OHSS pathogenetic factor. Exogenous levels of hCG are responsible for the early-onset OHSS, while the endogenous levels of hCG produced by the implanting fetus are responsible for the late OHSS. The hCG half-life is over 30 h, whereas LH half-life is less than 1.5 h (16, 17). The activation of LH receptors is significantly higher in case of hCG triggering, leading to a higher vascular permeability boosted by the secretion of the vascular endothelial growth factor (VEGF) (18).

The GnRH-a flare-up activity, in the antagonist-downregulated cycle setting, seems to be more closely mimicking the endogenous LH surge seen in natural cycles compared to hCG triggering (18, 19). The flare-up effect is caused by a temporary increase of LH/FSH secretion, but the surge has a shorter and different effect, comparing to hCG induction or natural cycles (19). Three independent studies by Cerrillo et al. (18), Lamb et al. (20), and Zelinski-Wooten et al. (21) reported lower steroid blood levels in case of GnRH-a triggering than those in hCG triggering cycles. It was found that the gene expression of different enzymes that take part in steroidogenesis at the moment of oocyte pickup was lower in cases of GnRH-a triggering (22). Significant reduction of VEGF expression in these patients may be a key factor in early OHSS prevention, such us a lack of hyperstimulated corpora luteal (8, 9, 16, 22).

### Is there an optimal dose for agonist triggering?

Nowadays, there are many types of GnRH-a available, and all of them have been thoroughly studied. Bodri et al. (9), Humaidan et al. (23), and Papanikolaou et al. (24) tested a single dose of triptorelin 0.2 mg**;** other researchers have explored other types and different administration protocols, such as buserelin 0.2 mg in intranasal administration (5), buserelin 0.5 mg (23, 25–27), leuprolide 1 mg (28, 29), and nafarelin (25). The main conclusion of their research was that there is no significant difference between different forms of GnRH-a with regard to effectiveness on ovulation induction. The data presented by Vuong et al. (30), in 2016, confirm the efficacy of triptorelin 0.2 mg vs. 0.3 and 0.4 mg based on the number of metaphase II (MII) oocytes and good transferable embryos.

Triptorelin in doses of 0.2 or 0.3 mg is the most used protocol for ovulation induction in egg donation programs (9, 31).

### Gonadotropin-releasing hormone triggering impact on oocyte number and embryo quality during oocyte donation

Embryo yield rates, during IVF, depend on multiple variables. However, the initial goal is to obtain a reasonable number of mature oocytes after final maturation triggering. Therefore, when replacing hCG with GnRH-a started, there was a lot of caution in the scientific community on whether the new triggering agonist regimen could efficiently replace the classical and lengthy use of hCG.

Initially, Humaidan et al. (23), in 2014, repeatedly reported no difference in the number of MII oocytes in both protocols in non-donor IVF cycles. Similarly, other authors have shown an equal number of mature oocytes between the classical hCG and agonist triggering (23).

Later studies that included oocyte donation cycles (32), fertility-preservation oncologic cycles (33, 34), and Pre implantation Genetic Testing for Aneuploidies (PGT-A) cycles (35) mentioned a significant difference and a higher number of MII oocytes and qualified transferable embryos in GnRH-a- vs. hCG-triggered cycles. Reddy et al. (33), in 2014, reported a higher number of MII oocytes (10.5 ± 5.1 vs. 7.7 ± 5.3, p = 0.002) and a higher number of cryopreserved embryos (7.7 ± 4.2 vs. 5.4 ± 3.8, p = 0.002) in oncologic patients triggered by GnRH-a. A large fertility preservation study by Pereira et al. (34), in 2017, confirmed higher effectiveness of GnRH-a triggering, demonstrating an increased number of MII oocytes and embryos frozen (11.8 vs. 9.9 with p = 0.04 and 9.2 vs. 6.4 with p < 0.001, respectively).

Some authors have raised concerns of empty follicle syndrome (EFS) being more frequent after GnRH-a triggering. EFS is a condition in which no oocytes are retrieved despite adequate ovarian stimulation and meticulous follicular aspiration. The incidence of EFS has been reported to range from 1% to 3.5% in literature (36). However, the data from large retrospective analyses, including 2,034 oocyte donation cycles, showed no statistically significant difference in the incidence of EFS between the GnRH triggering group (3.5%) and the hCG triggering group (3.1%) (37). An interesting case report suggests retriggering with hCG after failed GnRH-a triggering as a means of rescuing the cycle while retrieving oocytes that can lead to a normal pregnancy (38).

Regarding embryo yield, Galindo et al. (6), in 2009, studied 257 oocyte donor cycles and showed in both hCG and GnRH-a triggering groups no differences with regard to the number and status of embryos successfully transferred. Interestingly, Erb et al. (35), in 2010, based on his donation cycle retrospective research, concluded that GnRH-a triggering resulted in a higher number of all oocytes, MII oocytes, and embryos (p = 0.06) vs. the hCG triggering group (35).

### Gonadotropin-releasing hormone triggering impact on recipient’s pregnancy outcome during oocyte donation

A meta-analysis by Humaidan et al., in 2011, included four studies and concluded that the clinical pregnancy rates and delivery rates were similar between GnRH-a and hCG trigger in donor cycles (39). Three other studies have also reported similar implantation rates and clinical pregnancy rates (5, 7, 8). Moreover, very similar results were presented by Galindo et al. (6), in 2009, with clinical pregnancy, ongoing pregnancy, and life birth rates as primary outcomes in both triggering groups.

Furthermore, retrospective studies by Shapiro et al. (40), in 2007, and Bodri et al. (9), in 2009, in oocyte donor cycles reported very similar results. The effectiveness of GnRH-a triggering with respect to pregnancy rate was confirmed by the research of Castillo et al. (37), in 2012, showing a high pregnancy rate (79/123, 64.2%) per recipient. These results are in accordance with other publications (31, 32).

### Are ovarian hyperstimulation syndrome-free oocyte donation programs a reality?

Initial studies, administering GnRH-a triggering in the classical antagonist stimulation cycle setting, demonstrated total clinical OHSS prevention, including both moderate and severe forms of OHSS (34, 35).

In donor cycles, OHSS was not reported after GnRH-a triggering in seven clinical studies. However, in hCG triggering cycles, OHSS occurred between 4% and 17% of cases (5, 6, 8, 34, 41) (**Table 2**).

**Table 2:** OHSS rate in oocyte donation cycles comparing classical hCG triggering vs. GnRH-agonist triggering.

Agonist triggering hCG triggering			hCG triggering
Study	OHSSEvents	Donorsn (%)	OHSSEvents	Donorsn (%)
Acevedo, 2006 (5)	0	30	5	30 (17%)
Galindo, 2009 (6)	0	106	9	106 (8.5%)
Melo, 2009 (7)	0	50	2	50 (4%)
Sismanaoglou, 2009 (8)	0	44	3	44 (6.8%)
Bodri, 2009 (9)	0	1,046	13	1,031 (1.3%)
Streda, 2011 (10)	0	12	1	12 (8.3%)
Tsakiridis, 2020 (11)	0	80	3	80 (3.7%)
Total	**0**	**1,368 (0%)**	**36**	**1,353 (2.66%)**

Similarly, recently, Tsakiridis et al. (11) reported three cases of moderate OHSS (3.75%) after hCG triggering without any case of OHSS occurring after GnRH-a triggering (p = 0.25).

Despite an extremely low OHSS incidence after GnRH-a triggering, we cannot forget that the oocyte donors are, *a priori*, a group at higher risk for OHSS due to a significantly higher number of mature follicles on the day of triggering (42, 43). Based on no donor, but still high-risk patient group analysis, there are similar case reports published by Fatemi et al. (15), in 2014, Santos-Ribeiro et al. (44), in 2015, and Iorio et al. (45), in 2021, in patients who developed severe OHSS even after GnRH-a triggering.

The main limitation in the precise differential diagnosis of OHSS vs. post retrieval bleeding is also the lack of sufficient surgical data, which are available only in severe cases after laparoscopic confirmation of bleeding and the confusion concerning the definition of severe OHSS (41).

That is why we must always be vigilant, as severe OHSS after agonist triggering, although extremely rare, is still possible (46).

### Issues that should be taken into consideration

Although agonist triggering became the gold standard of ovulation induction especially in oocyte donors, precautions should still be taken.

1) LH levels on the day of triggering should be seriously considered, as deep downregulation might be present with LH levels <0.1 mIU/ml, and maybe in these cases, a dose of 0.2 triptorelin might not be sufficient to induce adequate gonadotropin release, and in turn, EFS might be encountered.

2) Clinicians should also consider low levels of LH during first days of hormonal stimulation, since some authors linked low follicular LH with poor oocyte collection cohort following GnRH-a triggering. They also noticed that in this group of poor, suboptimal responders, oral contraceptive priming might be associated with low LH starting levels (36).

3) Some reports indicated suboptimal hypophysis response, defined as a serum LH level lower than 12–15 mIU/ml measured 8–12 h after agonist triggering in young low-BMI donors. They postulated a possible connection of iatrogenically induced pituitary downregulation (LH <2 IU/L) caused by previous prolonged oral contraceptive pill (OCP) therapies with low LH levels on the day of GnRH-a triggering (47, 48). The only risk factor, significantly confirmed, was the long-term hormonal contraception [odds ratio (OR) 20.97; 95% confidence interval (CI), 5.29–83.14; p < 0.0001) rather than the short-term OCP use as part of a fertility pretreatment (49).

## Conclusions

1) The most popular type of GnRH-a used today is triptorelin in doses of 0.2 or 0.3 mg with confirmed effectiveness in ovulation induction.

2) Most of the authors confirm higher effectiveness of GnRH-a triggering compared to traditional hCG triggering, resulting in a higher number of both mature oocytes and good quality frozen embryos.

3) The implantation rates, clinical pregnancy rates, and life birth rates in recipients, presented in reviewed literature, were very similar in both triggering groups.

4) The main conclusion in terms of safety and the OHSS occurrence, drawn from retrospective studies and clinical trials, is that GnRH-a triggering almost completely eliminates early OHSS during and after stimulation in donation programs.

Further research is needed to increase the accuracy of the data in donation cycles.

## Author contributions

RN wrote and edited the manuscript, GM constructed the tables and edited the manuscript, NP edited the manuscript and looked into statistics, ET searched the literature and edited the manuscript, TC searched the literature and edited the manuscript, SK wrote the manuscript, FC reviewed and edited the manuscript, AM reviewed the manuscript and the statistics, EP wrote and edited the manuscript. All authors contributed to the article and approved the submitted version.

## Conflict of interest

The authors declare that the research was conducted in the absence of any commercial or financial relationships that could be construed as a potential conflict of interest.

## Publisher’s note

All claims expressed in this article are solely those of the authors and do not necessarily represent those of their affiliated organizations, or those of the publisher, the editors and the reviewers. Any product that may be evaluated in this article, or claim that may be made by its manufacturer, is not guaranteed or endorsed by the publisher.

## Abbreviations

ART, assisted reproductive technology; IVF, *in vitro* fertilization; OHSS, ovarian hyperstimulation syndrome; GnRH, gonadotropin-releasing hormone; GnRH-a, gonadotropin-releasing hormone agonist; hCG, human chorionic gonadotropin; LH, luteinizing hormone; FSH, follicle-stimulating hormone; MII, metaphse II.
