# Supplementary figures and images for: Agonist triggering in oocyte donation programs—Mini review
Source: Front Endocrinol (Lausanne). 2022 Aug 26;13:838236. doi: 10.3389/fendo.2022.838236 (PMC9462512; doi:10.3389/fendo.2022.838236)

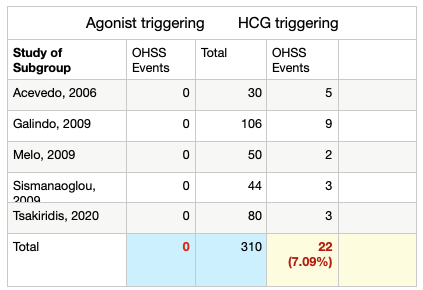

Supplement: Supplementary file 1 [file Image_1.png]
